# Time-Resolved Extracellular Matrix Atlas of the Developing Human Skin Dermis

**DOI:** 10.3389/fcell.2021.783456

**Published:** 2021-11-26

**Authors:** Mansheng Li, Xiao Li, Binghui Liu, Luye Lv, Wenjuan Wang, Dunqin Gao, Qiyu Zhang, Junyi Jiang, Mi Chai, Zhimin Yun, Yingxia Tan, Feng Gong, Zhihong Wu, Yunping Zhu, Jie Ma, Ling Leng

**Affiliations:** ^1^ State Key Laboratory of Proteomics, Beijing Proteome Research Center, National Center for Protein Sciences (Beijing), Beijing Institute of Life Omics, Beijing, China; ^2^ Institute of NBC Defense, Beijing, China; ^3^ Department of Dermatology, Chinese PLA General Hospital, Beijing, China; ^4^ Stem Cell and Regenerative Medicine Lab, State Key Laboratory of Complex Severe and Rare Diseases, Department of Medical Science Research Center, Translational Medicine Center, Peking Union Medical College Hospital, Chinese Academy of Medical Sciences and Peking Union Medical College, Beijing, China; ^5^ Department of Plastic and Reconstruction Surgery, Chinese PLA General Hospital, Beijing, China; ^6^ Department of Stem Cell and Regenerative Medicine Laboratory, Institute of Health Service and Transfusion Medicine, Beijing, China; ^7^ Basic Medical School, Anhui Medical University, Anhui, China

**Keywords:** decellularization, extracellular matrix, matrisome, skin development, skin aging

## Abstract

Skin aging is a physiological issue that is still relatively poorly understood. Studies have demonstrated that the dermal extracellular matrix (ECM) plays important roles in skin aging. However, the roles of the changes in ECM characteristics and the molecules that are secreted to the extracellular space and are involved in the formation of the dermal matrix from birth to old age remain unclear. To explore the way in which the ECM microenvironment supports the functions of skin development across different age groups is also poorly understood, we used a decellularization method and matrisome analysis to compare the composition, expression, and function of the dermal ECM in toddler, teenager, adult, and elderly skin. We found that the collagens, glycoproteins, proteoglycans, and regulatory factors that support skin development and interact with these core ECM proteins were differentially expressed at different ages. ECM expression markers occurring during the process of skin development were identified. In addition, our results elucidated the characteristics of ECM synthesis, response to skin development, and the features of the ECM that support epidermal stem cell growth *via* the basement membrane during skin aging.

## Introduction

The human skin is a complex organ comprising a range of tissues that act in harmony to create a protective barrier against environmental stresses such as heat, solar ultraviolet light, irradiation, and pathogens, and regulates the body’s temperature and degree of water loss (Quan and Fisher, 2015). The human skin consists of two major layers. The upper layer, or *epidermis*, is a multilayered epithelium that continually undergoes terminal differentiation. Underneath the *epidermis* lies the dermis, which is enriched with dermal fibroblasts, vascular connective tissue, and dense extracellular matrix (ECM). Dermal collagen constitutes the bulk of the skin, making up 90% of the skin by dry weight (Uitto, 1986). The mechanical properties and functioning of the skin depend on the composition, structure, and organization of the dermis (Haake et al., 2001).

The human skin undergoes a natural aging process in response to intracellular and external stresses, and damage from environmental sources (Naylor et al., 2011). A study of 1,713 American women aged over 50 years highlighted the psychological suffering associated with aging (Hofmeier et al., 2017). Aging is generally believed to be related to the alterations of the dermal ECM (Gunin et al., 2010). On the basis of their structural and functional features, ECM components are divided into the “core matrisome”, made up of collagens, proteoglycans, and glycoproteins, and “matrisome-associated” components, comprising ECM-bound proteins and carbohydrates. These carbohydrates include glycosaminoglycan (GAG) chains, which are not linked to a core protein but are bound by anionic charge binding to proteins. They include regulatory signals and secreted factors bound to one or more core matrisome components (termed the “matrisome”) (Naba et al., 2016). During the first 10 years of childhood, the dermis develops at the cellular level (Gunin et al., 2010). In fetuses, *in utero* and toddler, the skin transiently has the functional characteristic of wound healing with only slight scarring (Krejčí et al., 2015). Although there have been several studies on the ECM in young and aging skin, including investigations of the roles of collagens, the elastic fiber network, proteoglycans, and GAGs, (Ge et al., 2020; Mccabe et al., 2020), the composition and function of the ECM over the course of skin development remain unclear.

Most previous findings have been based on the expression of ECM proteins in cells or whole tissues, (Kaur et al., 2019; Ewald, 2020; Ge et al., 2020), rather than those secreted from cells, due to a lack of effective methods with which to obtain the complete bioactive scaffold while preserving the ECM components. We previously developed a method for skin decellularization and used it to investigate differences in the composition and expression of whole tissue ECM and secreted ECM (Leng et al., 2020; Liu et al., 2020). Our strategy facilitates the application of tissue engineering principles to skin bio matrix scaffold materials, promising to accelerate, and enhance tissue regeneration. Using this method, we identified the extracellular matrisome composition of human, pig, and rat skin. On the basis of the conclusions drawn from this work, we have analyzed the pathological features of the matrisome in skin keloids, providing a useful model for the diseases and identifying potential targets for therapy (Zhang et al., 2021). In this study, we used the same method, combining a quantitative proteomics approach to the analysis of ECM composition in skin decellularized bioscaffolds of different ages and aiming to establish a new standard of “young and active” skin ECM composition and to discover new clues regarding tissue engineering for skin regeneration.

## Materials and Methods

### Sample Preparation

A total of ten skin tissue samples from healthy individuals of four different ages, i.e., toddler (1–3 years old), teenager (8–18 years old), adult (30–50 years old), and elderly (>60 years old), were used in this study. All skin samples were collected from the abdomen, thigh, or back of the donors, and provide by the Department of Dermatology, PLA General Hospital, China. Written informed consent was obtained from all the participants. Detailed characteristics of these healthy individuals are provided in Supplementary Table S1. This study was approved by the Medical Ethics Committee of Chinese PLA General Hospital (NO. S2018-123-02).

### Preparation of Decellularized Skin Scaffolds

A 3- to 6-mm-thick segment of skin tissue was cut using an electric skin picker. Skin tissues were rinsed with cold phosphate-buffered saline (PBS), followed by delipidation using phospholipase A2 combined with sodium deoxycholate for 4 h in a shaker at 37°C until the tissue segments became oyster white. The surface of the skin was scraped gently and carefully using the back of a scalpel to remove the *epidermis*. The decellularized dermal samples were placed in sterilized 1.5 ml microcentrifuge tubes. Then, samples were rinsed using 3.4 M NaCl for 1 h followed by a final wash with PBS containing nucleases (10 μg/ml DNase, 5 μg/ml RNase) at 37°C for 1 h. Finally, the decellularized scaffolds were flash-frozen for proteomics analysis.

### Protein Extraction, Digestion, and Liquid Chromatography–Tandem Mass Spectrometry (LC–MS/MS) Analysis

Decellularized human skin scaffolds were homogenized, and proteins were extracted using 1 ml Protein Extraction Reagent Type 4 (Sigma-Aldrich) for every 125 mg of pulverized tissue. After centrifugation at 4°C and 14,000 × *g* for 10 min, the supernatants were reduced by adding 1 × protease inhibitors tributylphosphine to a final concentration of 5 mM, followed by vortexing for 10 min at room temperature. After centrifugation at room temperature and 14,000 × *g* for 30 min, supernatants were transferred to a clean tube and stored at −80°C. For peptide extraction, 25 μg samples (from the supernatants) were solubilized in 10 mM DTT at 37°C for 4 h. Samples were added with 1 M IAA and kept in the dark for 1 h. All samples were collected, and the suspension was removed after centrifugation for 12,000 × *g*. Thereafter, 100 μl UA was added and the suspension was removed after centrifugation twice. NH_4_HCO_3_ (50 mM) was added to the samples, and the supernatants were removed after centrifugation at room temperature and 12,000 × *g* for 5 min. A final digestion was performed by incubating with trypsin at a ratio of 1:50 enzyme/substrate, at 37°C overnight. After centrifugation at 14,000 × *g* for 30 min, the supernatants were transferred to clean tubes for the LC–MS/MS analysis. The peptide mixtures were analyzed using Q-Exactive mass spectrometer (Thermo Fisher Scientific) equipped with an Easy-nLC nanoflow LC system (Thermo Fisher Scientific).

### MS/MS Data Identification and Bioinformatics Analysis

Raw MS files were analyzed using the MaxQuant software (version 1.6.5.0) (Cox and Mann, 2008) against the human UniProt database (https://www.uniprot.org/, accessed on May 13th, 2020) and contaminants database. Peptide identification was performed using a precursor mass tolerance of up to 4.5 ppm and a fragment mass tolerance of 20 ppm. Cysteine carbamidomethylation was set as the fixed modification, and N-terminal acetylation and methionine oxidations were used as variable modifications. Up to two missed cleavages were allowed, and trypsin was set as the enzyme specificity. Automatic target and reverse database searches were used, and a false discovery rate of 1% at both the peptide and protein levels was allowed. Protein quantification was performed based on the intensity-based absolute quantification method (Schwanhausser et al., 2011) embedded in MaxQuant.

All bioinformatics analyses were performed using the R language and statistical environment, version 3.6.3. The quantification values of identified proteins were normalized by taking the fraction of total, followed by multiplication by 10^6^. To test for significant differences in the expression of proteins between the four different age groups, one-way ANOVA (analysis of variance), or Kruskal–Wallis tests were performed. The R package clusterProfiler (version 3.12.0) (Yu et al., 2012) was used to annotate the identified proteins according to the Biological Processes, Cellular Components, and Molecular Functions defined by the Gene Ontology (GO) (Ashburner et al., 2000). KEGG pathway analysis was also performed (Ogata et al., 1999) (https://www.kegg.jp/kegg/pathway.html). Tissue expression information regarding the significant proteins was retrieved and integrated from the online tool DAVID (https://david.ncifcrf.gov/) (Huang Da et al., 2009). ECM proteins, including six categories of core ECM (collagens, proteoglycans, and ECM glycoproteins) and ECM-associated proteins (ECM regulators, ECM-affiliated proteins, and secreted factors), were annotated using MatrisomeDB (http://matrisomeproject.mit.edu/) (Naba et al., 2016). Heat maps of the ECM protein quantitation values were constructed using the R package ComplexHeatmap (version 2.0.0) (Gu et al., 2016). The basement membrane (BM) located ECM proteins were confirmed by the GO’s Cellular Components annotation retrieved from DAVID. We also used the circlize package (version 0.4.11) (Gu et al., 2014) to circularly visualize the upregulation and downregulation of ECM proteins in human skin dermis with aging.

### Immunochemical Staining

Samples were fixed in 4% formaldehyde overnight at 4 °C and then processed using a dehydration gradient. After tissues were embedded in paraffin, sections were cut to a thickness of 4 μm for hematoxylin and eosin staining, immunohistochemistry and immunofluorescence analysis. The sections were deparaffinized and heated with a microwave to boil for at least 12 min with antigen retrieval buffer, removed by endogenous catalase in 0.3% H_2_O_2_ for 30 min after cooling. Then, sections were blocked with normal horse serum in Tris-buffered saline for 1 h and blocked with Avidin/Biotin Blocking Kit, stained with antibodies ab11575 (anti-laminin antibody LAMC3, Abcam, dilution: 1:200), ab6586 (COL4A1, Abcam, dilution: 1:1,000), 13530-1-AP (NID2, Proteintech, dilution: 1:200), ab97779 (MMP2, Abcam, dilution: 1:400), and 11060-1-AP (ANXA5, Proteintech, dilution: 1:200) overnight at 4°C. After staining with a secondary antibody, the sections were colored. For immunofluorescence, skin sections were analyzed for COL6A1 and COL12A1 expression using antibodies against 17023-1-AP (COL6A1, Proteintech, Sankt Leon-Rot, Germany, dilution: 1:200) and 19727-1-AP (COL12A1, Proteintech, dilution: 1:200), and SLPI against sc-373802 (SLPI, Santa Cruz Biotechnology, Inc., United States). Samples were then incubated with secondary antibodies (SA00009-2, Proteintech) for 1 h at room temperature and counterstained with DAPI. Images were taken at ×20 and ×40 magnification and analyzed using Volocity Demo (×64) (PerkinElmer, Waltham, MA).

## Results

### Skin Proteome Profile of Different Ages

**FIGURE 1:**
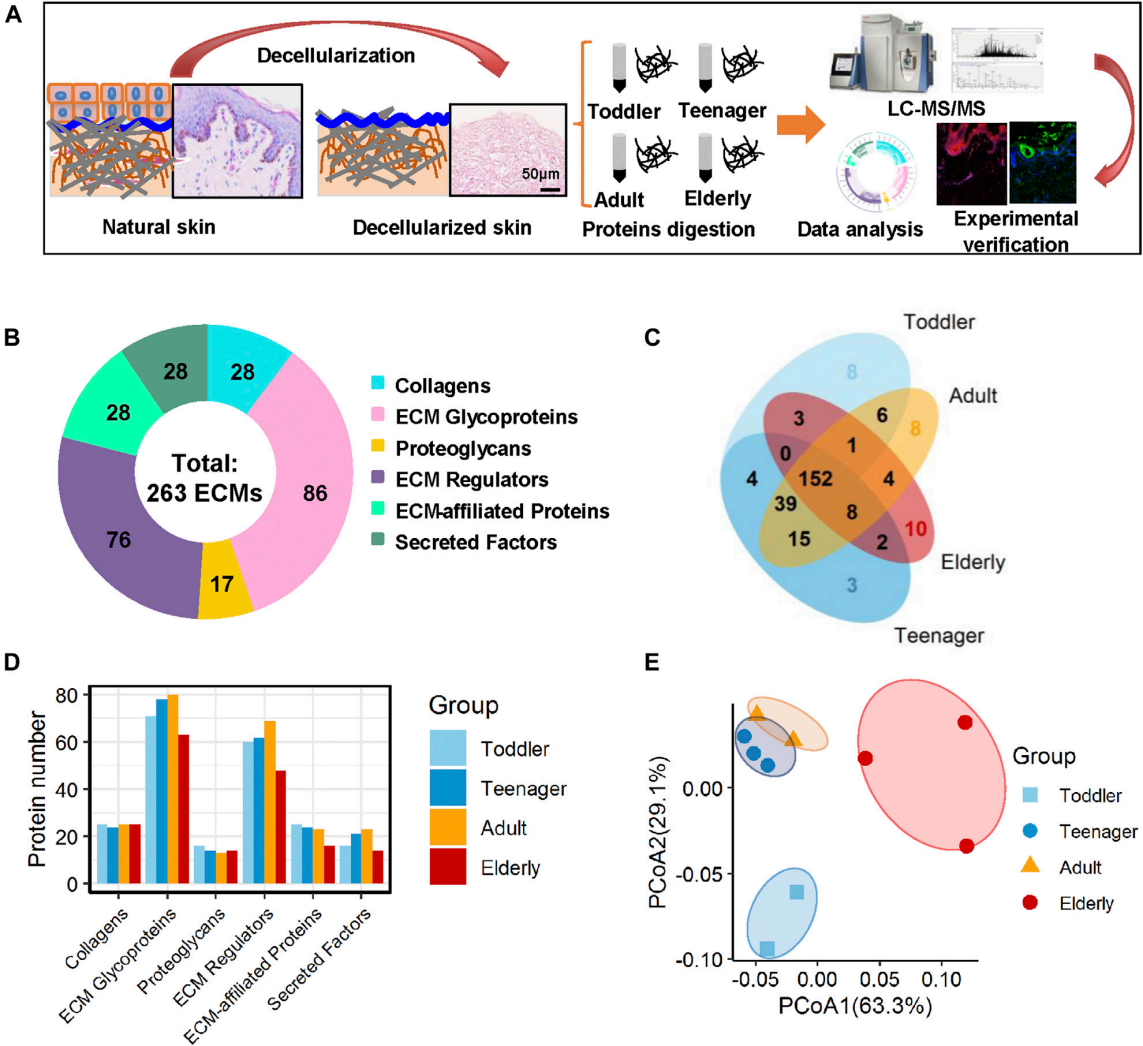
Matrisome profile of the human skin dermis. **(A)** General workflow of the whole experimental design, including decellularization, mass spectrometry-based quantitative proteomics, bioinformatics analyses, and experimental verification. **(B)** Six types of ECM proteins (collagens, ECM glycoproteins, proteoglycans, ECM regulators, ECM-affiliated proteins, and secreted factors) expressed at four ages (toddler, teenager, adult, and elderly). Pie charts represent the proportion of the six components (collagens, ECM glycoproteins, proteoglycans, ECM regulators, ECM-affiliated proteins, and secreted factors) that make up the matrisome in the human skin dermis. **(C)** Overlap of ECM proteins in the skin scaffolds of four ages (toddler, teenager, adult, and elderly). **(D)** Histogram shows the number of the six ECM components identified at four age stages. **(E)** PCoA analysis of the samples at four ages (toddler, teenager, adult, and elderly) based on their ECM profile. The technical repeats were produced for each sample, represented by different colored points in the figure.

**FIGURE 2:**
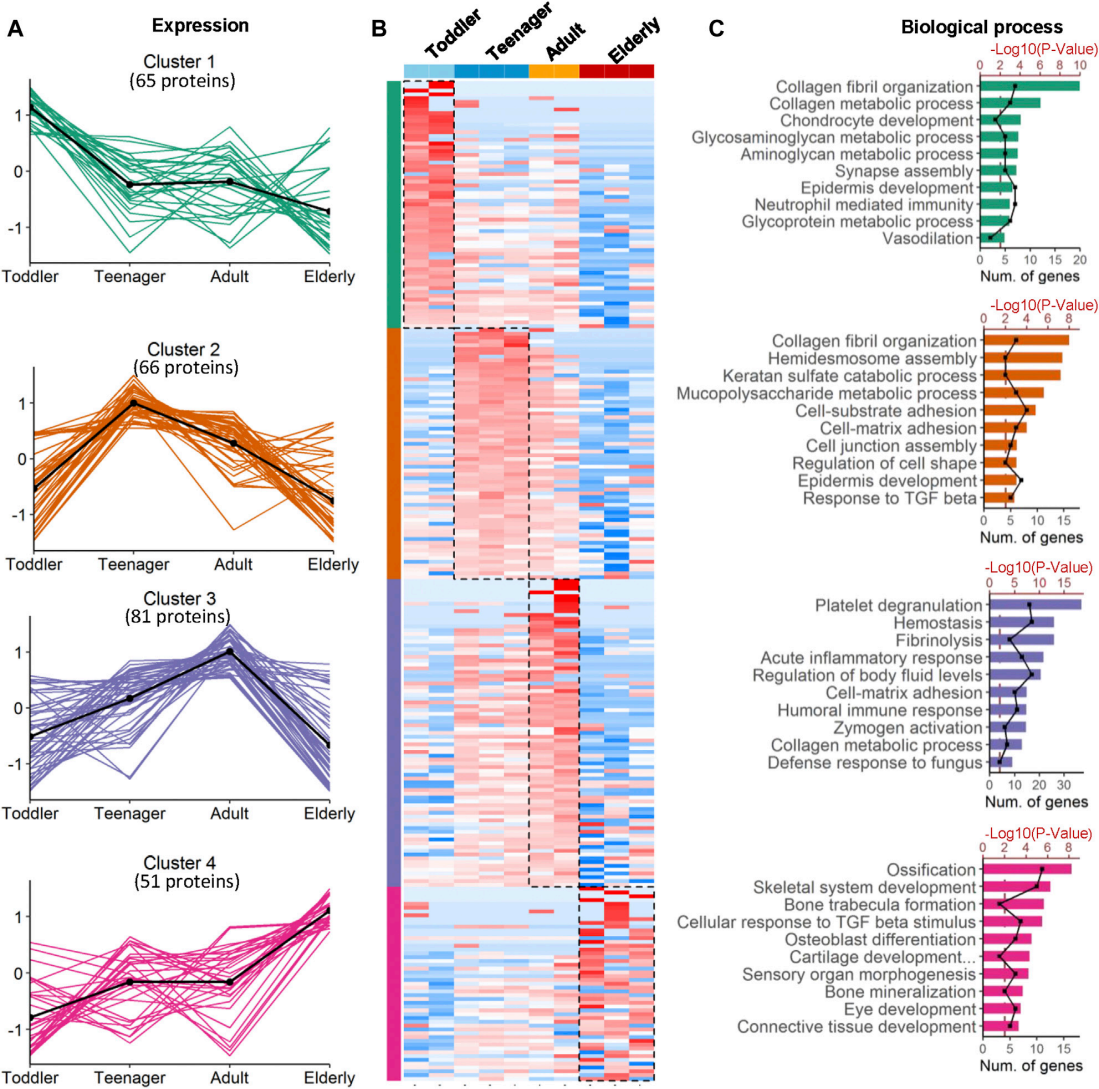
Coexpression analysis of age-specific ECM protein modules. **(A)** Four protein modules revealed ECM specificity based on age stage. Cluster 1−4 are the protein groups highly expressed in toddler, teenager, adult and elderly, respectively. **(B)** Heatmap analysis of ECM identified in samples from skin bioscaffolds of four ages (toddler, teenager, adult, and elderly) according to log_2_ normalized protein intensity. Red and blue boxes indicate proteins with increased and decreased abundance, respectively. Green, purple, and rose bars correspond to the proteins enriched in skin bioscaffolds from toddler, teenager, adult, and elderly. **(C)** Clusters of proteins associated with similar biological process were grouped according to the degree of enrichment.

To investigate the ECM composition of different ages, decellularized skin scaffolds were obtained using the decellularized method established by our group previously (Leng et al., 2020; Liu et al., 2020; Zhang et al., 2021). Especially, the decellularized skin scaffolds remained the BM structure (Figure 1A and Supplementary Figure 1A). Next, Raman spectroscopy was used to analyze skin tissues of different ages and found that the decellularized samples, including the BM and dermis (DM), could be easily distinguished from native skin tissues (Supplementary Figure S1B–E). Quantitative proteomics is an important method for the identification of the ECM components secreted into, and located in, the extracellular space (Leng et al., 2020; Liu et al., 2020). To investigate the molecular basis of skin aging, we used a quantitative proteomics approach to analyze the composition of the ECM in skin decellularized bioscaffolds of different ages. The overall workflow is shown in Figure 1A. We used these data to establish a secreted “ECM map” and a developmental pattern of the dermal ECM during skin development (Supplementary Figure S2A–C). The pairwise Pearson’s correlation coefficients of repeat experiments with the samples from the same age group show good reproducibility, with a high positive correlation (correlation coefficient: 0.69–0.94) (Supplementary Figure S2B). Annotated by the MatrisomeDB, ECM proteins are divided into “core matrisome” proteins including collagens, glycoproteins and proteoglycans, as well as “matrisome associated” proteins including ECM-affiliated proteins, ECM regulators, and secreted factors. A total of 263 ECM proteins of six types were identified in the study, with 213, 223, 233 and 180 ECM proteins in toddlers, teenager, adult, and elderly skin tissues respectively (Figures 1B,C, Supplementary Figure S2C and Supplementary Table S2). There were 152 ECMs commonly identified in skin tissues of all four age stages with an overlap rate only about 57.8% (Figure 1C). Besides, ECM glycoproteins, ECM regulators, ECM-affiliated proteins, and secreted factors in the elderly were present at lower levels than those in the other three age groups (Figure 1D). Principal coordinates analysis (PCoA) was performed to explore the similarities and dissimilarities of the samples in the four age modules based on the ECM characteristics, and the results suggest that the samples from the four groups differed greatly (Figure 1E). To identify representative ECM characteristics at different ages and understand their biological significance, we performed differential expression analysis on all ECM at the four different ages (Figures 2A,B and Supplementary Table S2). Biological process analysis revealed that the skin of toddlers was mainly associated with collagen fibril organization, glycosyl metabolism, and chondrocyte and *epidermis* development. Skin from the teenager stage mainly exhibited hemidesmosome assembly, cell-matrix adhesion, cell junction assembly, and response to growth factors. For adults, the highly expressed proteins of skin were mainly associated with acute inflammatory responses, wound healing, and defense against fungi. Elderly skin mainly expressed protein concerned with the process of ossification, bone mineralization, and connective tissue development (Figure 2C), which may be one of the reasons for the lack of mechanical elasticity in elderly skin. These results indicated that the ECM matrisome microenvironment at the four ages reflects the developmental characteristics of the skin tissue.

### Skin Core Matrisome Features at Different Ages

**FIGURE 3:**
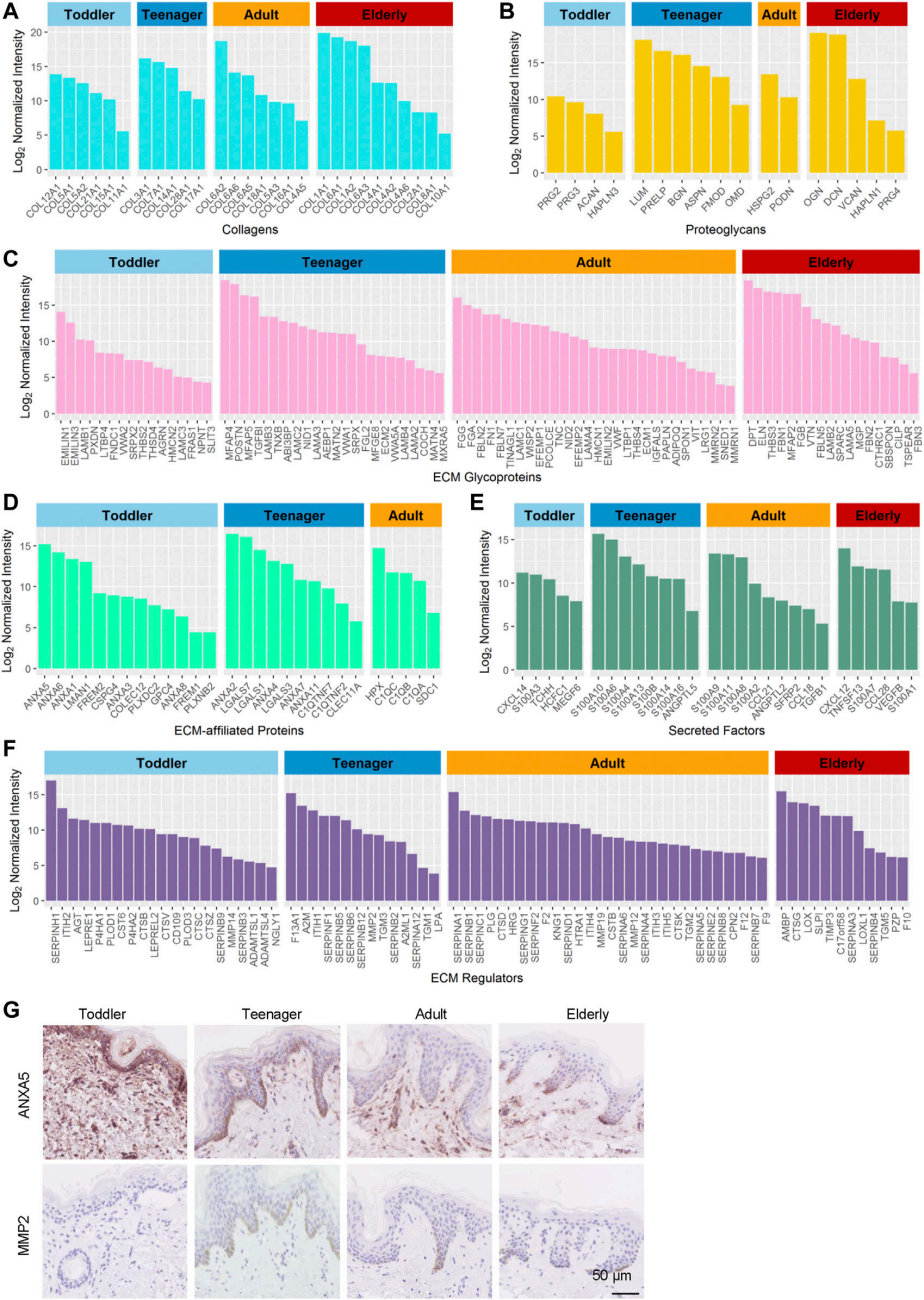
Highly expressed proteins of the six ECM components in the human skin dermis at four ages (toddler, teenager, adult, and elderly). **(A–F)** Histograms show highly expressed proteins of the collagen ECM, proteoglycan ECM, ECM glycoprotein, ECM-affiliated protein, secreted factor ECM, and ECM regulator, respectively. **(G)** Immunohistochemistry of ANXA5 and MMP2 in the skin tissues of toddler, teenager, adult, and elderly (scale bar represents 50 μm).

Collagens are the principal structural component of the cross-linked networks of the ECM. During skin development, the proportions of the fibril-associated collagens COL12A1, COL21A1 and COL11A1, (Ricard-Blum, 2011), as well as the BM collagens COL5A1/2 and COL15A1, were higher in toddler bioscaffolds than in any other stage of life (Figure 3A and Supplementary Table S3). The fibril-forming collagen COL3A1, the fibril-associated collagen COL14A1, and the BM collagens COL7A1, COL28A1, and COL17A1 were highly expressed in teenager bioscaffolds, whereas the short-chain collagen COL6A2/5/6, the fibril-forming collagen COL5A3, the micro fibril collagen COL16A1, and the BM collagens COL18A1 and COL4A5 were highly expressed in adult bioscaffolds. The skin of the elderly is generally believed to have decreased levels of collagens. (Quan and Fisher, 2015). However, we found that the short-chain collagens COL6A1, the fibril-associated collagens COL1A1/2, and the BM collagens COL4A2/6, COL2A1, and COL8A1, as well as a cartilage-specific collagen, COL10A1, were in elder bioscaffolds.

Proteoglycans are interspersed among collagen fibrils in the ECM and provide structural strength. They can store and release biologically active soluble signals by binding to sulfated carbohydrate chains of GAGs (Farach-Carson et al., 2014). Our results revealed that toddler bioscaffolds showed the highest expression of the hyaluronic acid binding proteoglycans, including ACAN and HAPLN3 (Figure 3B), which may generate a large osmotic swelling pressure, conferring skin stiffness and resistance to deformation (Juhlin, 1997), as well as skin hydration and viscoelasticity (Hernández et al., 2011). A small leucine-rich proteoglycans (LUM), and two bone-related ECMs (ASPN and OMD) were found highly expressed in teenager bioscaffolds. Five proteoglycans, including BGN, DCN, VCAN, HAPLN1, and PRG4, were found highly expressed in bioscaffolds from elderly donors. Proteoglycans can also influence the rate of assembly, size, and structure of collagen fibrils (Schonherr and Hausser, 2000; Kadler et al., 2008), indicating that highly expressed age-specific proteoglycans can affect the skin by regulating collagens.

Glycoproteins are major components of the noncollagenous core of the matrisome components of the skin ECM, regulating cell proliferation, migration, differentiation, and wound healing (Pankov and Yamada, 2002; Rousselle et al., 2019). Our results indicated that most of the highly expressed glycoproteins in toddler bioscaffolds were related to the member of the elastin microfibril family (EMILIN3), regulators of transforming growth factor beta (THSD4 and LTBP4), and tissue development (NPNT and FRAS1) (Figure 3C). Highly expressed glycoproteins in teenager skin were involved in elastic fiber assembly (MFAP4 and TNXB) and the formation of filamentous networks (MATN4). Angiogenesis-associated ECM proteins (ADIPOQ, THBS4, MMRN1, and MMRN2), neuronal development (FBLN7) were highly expressed in adult skin. In elderly skin, highly expressed glycoproteins were involved in bone mineralization (MGP) and response to transforming growth factor beta (FBN1 and CILP). These results indicated that the mechanical and functional properties provided by the different compositions of these core matrisomes at different ages may determine skin aging.

### Regulatory Matrisomes Are Essential for Skin Function

In addition to the core matrisome proteins, the composition and regulation of matrisome-associated proteins, such as ECM-affiliated proteins, regulators, and secreted factors, were studied in the aging dermis. Our results revealed that 23, 20, 15, and 14 matrisome-associated proteins, most of which are regulators of cell attachment and growth (Martin et al., 1984; Naba et al., 2012), were highly expressed in toddler, teenager, adult, and elderly bioscaffolds, respectively (Supplementary Table S3). In toddler skin, the highly expressed matrisome-associated proteins included placental anticoagulant proteins (ANXA5/8) and ECM associated proteases (CTSV, ADAMTSL1/4, PLOD3, P4HA1/2, and NGLY1) (Figures 3D–F). Meanwhile, the placental anticoagulant protein (ANXA4), proteins specifically expressed in keratinocytes (LGALS3/7 and TGM1/3), C1q tumor necrosis factor-related proteins (C1QTNF2/C1QTNF7), cell cycle and differentiation regulators (S100A10/14 and S100B), and protease inhibitors (SERPINB12, SERPINF1, and SERPINA12) are highly expressed in teenager skin. In adult scaffolds, the matrisome-associated proteins with high expression level were mainly associated with blood coagulation (KNG1 and F12), inflammation (SDC1, S100A2/8, CCL18/21, and TGFB1), fibrinolysis (HRG), hyaluronic acid regulation (ITIH4/5), and the inhibition of proteases (SERPINB8, SERPINC1, SERPINF2, and SERPINE2). In older scaffolds, those proteins were mainly involved in inflammation (CXCL12, S100A7, TNFSF13, CCL28, VEGFB, S100A1, AMBP, and SLPI) and aging (LOXL1). Among these proteins, the protein ANXA5 with high expression in toddler and MMP2 with high expression in teenager were selected for the verification of the expression patterns during aging using immunohistochemistry staining (Figure 3G). These results indicated that anticoagulant and cell development-associated matrisome-associated components were highly expressed in toddler and teenager. Wound healing and aging associated matrisome-associated components were mainly expressed in adult and elder bioscaffolds.

### Functional Matrisome Composition of EpSCs Niche

**FIGURE 4:**
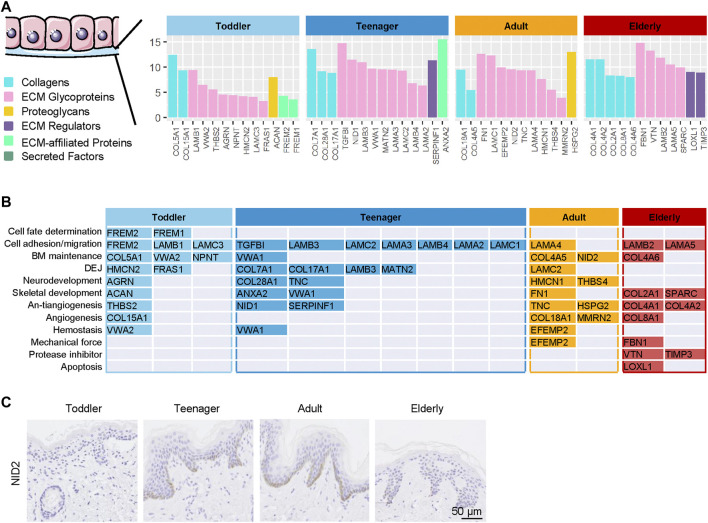
| Highly expressed basement membrane (BM) proteins in the six ECM components in the human skin dermis from four ages (toddler, teenager, adult, and elderly). **(A)** Six types of ECM proteins of BM (collagens, ECM glycoproteins, proteoglycans, ECM regulators, ECM-affiliated proteins, and secreted factors) expressed at four ages (toddler, teenager, adult, and elderly). **(B)** Functional analysis of BM located ECM from four ages (toddler, teenager, adult, and elderly). **(C)** Immunohistochemistry of NID2 in the skin tissues of toddler, teenager, adult, and elderly (scale bar represents 50 μm).

Aging is related not only to the dermal ECM but also to the state of Epidermal stem cells (EpSCs) (Stern and Bickenbach, 2007). The BM is a major component of the natural stem cell niche of basal EpSCs and provides necessary complex stimuli that affect the behavior of basal cells (Leng et al., 2020). BM components in samples from the four age categories were analyzed to evaluate how they support EpSC function throughout skin development. Our results revealed that 13, 14, 12, and 12 BM ECM proteins were specifically highly expressed in the skin of toddler, teenager, adult, and elderly, respectively (Figure 4A and Supplementary Table S4). Toddler scaffolds were mainly enriched in ECM proteins involved in the process of cell fate determination (FREM1/2), cell adhesion and migration-related ECM (FREM2 and LAMC3), and BM maintenance ECM (COL5A1, VWA2, and NPNT) (Figure 4B). Teenager bioscaffolds were mainly enriched in the proteins of cell fate determination-related ECM (LAMA3, LAMB3/4, and LAMC2) and dermal–epidermal junctions (DEJ) (COL7A1, COL17A1, LAMB3, and MATN2). Among these, mutations in COL7A1 and COL17A1 were associated with dystrophic epidermolysis bullosa (Burgeson, 1993; Dang and Murrell, 2008). In addition, COL17A1 is an important anchoring fibril related to the hemidesmosome complexes, but it was also identified as a novel EpSC marker (Bilousova and Degregori, 2019; Liu et al., 2019). Neurodevelopment and antiangiogenesis-related ECM were relatively abundant in teenager, adult, and elderly scaffolds. We found two protease inhibitors (VTN and TIMP3) and an apoptosis-related ECM protein (LOXL1) in elder scaffolds. These results indicated that the ECM composition of the BM, which is in direct contact with EpSCs, differs according to the stage of skin development. NID2, a high expressed protein in adult, was selected for the verification of the expression patterns during aging using immunohistochemistry staining (Figure 4C). These different EpSCs stimuli may explain the differences in EpSCs activity at different ages.

### Regulatory Pattern of ECM in the Process of Aging

**FIGURE 5:**
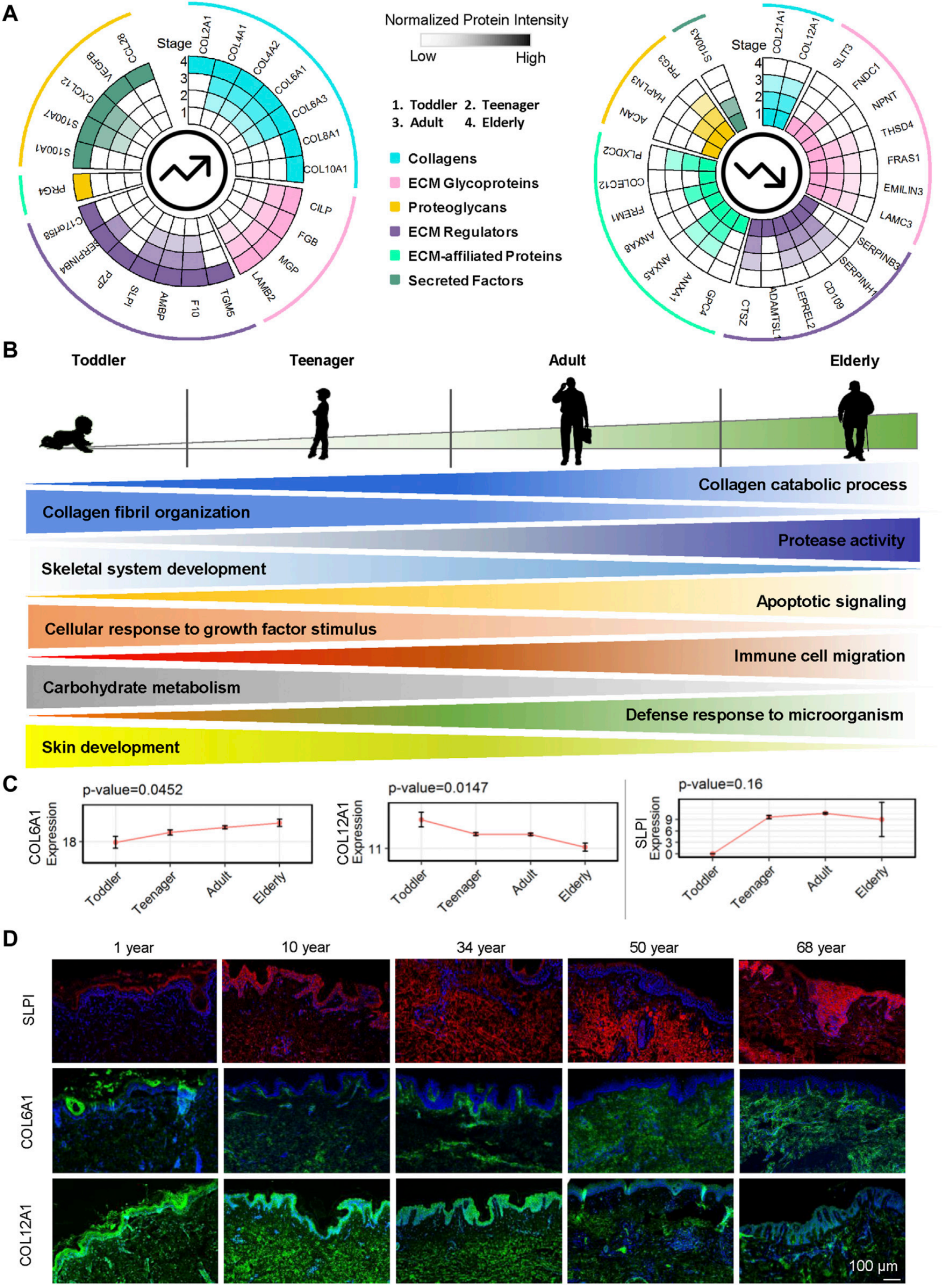
Roles of ECM proteins in human dermal development. **(A)** Upregulated and downregulated ECM proteins in the human skin dermis at four ages (toddler, teenager, adult, and elderly). Each circle represents an age stage. Blue, pink, yellow, purple, green, and cyan lines correspond to the six components (collagens, ECM glycoproteins, proteoglycans, ECM regulators, ECM-affiliated proteins, and secreted factors) of the skin ECM. **(B)** Schematic representation of dermal ECM function evolution during human skin development and aging. **(C)** Expression of COL6A1, COL12A1, and SLPI in skin scaffolds of four ages (toddler, teenager, adult, and elderly). **(D)** Immunofluorescence of SLPI, COL6A1, and COL12A1 identified in the human skin dermis (scale bar represents 50 μm).

Here we focused on the ECM proteins that gradually increased or decreased from toddler to elderly. Markers of epidermal development from EpSCs to mature keratinocytes have been well studied (Gonzales and Fuchs, 2017), whereas ECM markers for dermal development have not. We identified 24 ECM proteins upregulated with age and 26 ECM proteins downregulated with age among the four age groups (Figure 5A and Supplementary Table S2). Our results indicated that during skin development, ECM expression of collagen catabolic processes, protease activity, apoptotic signaling, immune cell migration, and defense response to microorganisms increased gradually (Figure 5B and Supplementary Figure S3A). These findings indicted that cell vitality, the ability to resist external microorganisms, and ECM remodeling are decreased as skin ages, resulting in increased susceptibility to skin infection and injury. Aging and relaxation of the skin appear to increase microorganism invasion and inflammatory factor production and enable the skin to continue undergoing wound healing, resulting in the formation of scars, which may also cause accelerated skin aging. With age, collagen fibril organization, skeletal system development, cellular response to growth factor stimulus, carbohydrate metabolism, and skin development decreased gradually (Figure 5B and Supplementary Figure S3B). For example, COL6A1, SLPI, and AMBP were gradually upregulated whereas COL12A1, COL21A1, FRAS1, ANXA5, and CD109 were downregulated over the course of the lifetime (Figure 5C and Supplementary Figure S4). COL6A1, COL12A1, and SLPI were selected for the verification of the expression patterns during aging using immunofluorescence staining (Figure 5D). COL6A1 was found to be specifically located around the BM of the epidermal–dermal junction in 10-year-olds, and then, with increasing age, its level of expression gradually increased in the dermis. By contrast, the collagen bundles of COL12A1 were highly expressed in 10- and 34-year-old dermis, and their expression decreased significantly with age.

## Discussion

Developmentally, extracellular matrices, composed of core ECM and ECM associated regulators, provide a tissue framework and cell niche that provides a micro- and macroarchitecture to help in driving cell behavior and function. Decellularized ECM derived from organs or tissues using physical and chemical treatments were used to study cell-matrix interactions based on their characteristics of complex composition, formation of vascular networks, and unique, tissue-specific architecture. Naturally derived biomaterials have physical and chemical effects that collectively form a foundation that can be used for the bioengineering of tissue bionic construction and that directly promotes tissue regeneration through secreted factors (Sadtler et al., 2016). Better understanding of ECM composition should lead to increases in their use *in vitro* and *in vivo* and should lead to the development of more robust tools for bioengineering and regenerative medicine strategies.

Alterations to the dermal ECM composition and organization are major drivers of aging of human skin. The biology of human skin aging can be viewed largely as a disorder of the dermal matrisome. However, our current knowledge regarding the impact of aging on the human skin matrisome is very limited. In this study, we used a tissue engineering method combined with a quantitative proteome technique to analyze the ECM composition and the function of human skin tissues during aging. Collagen is the main component of human skin. We found that the BM collagens were the main collagens expressed in an age-specific manner in toddler and teenager skin and short-chain and fibril-associated collagens were more abundant in adult and elderly skin. The proportions of these collagens may determine, to a great extent, the different physical structure and mechanical properties of skin at different ages. For example, we found that COL12A1 was highly expressed in toddler dermal skin, and as the skin aged, COL12A1 almost disappeared from the skin, indicating its important role in the physical support of the skin structure. Proteoglycans associated with skin stiffness and resistance to deformation were most abundant in toddler skin, and elastin micro fibril related glycoproteins were found in high levels in toddler and teenager skin, an observation which may explain why young people have more elastic skin.

ECM factors associated with skin development, such as growth factors and tissue developmental glycoproteins, were enriched in toddler skin. Keratinocytes specifically expressed in ECM were mainly in teenager skin. Epidermal stem cell proliferation and angiogenesis-related proteoglycans, and angiogenesis and neuronal development-related glycoproteins were found mainly in adult skin. We found that wound healing associated ECM, such as fibrinogen-related glycoproteins and blood coagulation-related regulators, as well as immunocyte stimulation and inflammation-related proteoglycans, was enriched in adult skin. Ossification and fibrosis-related glycoproteins and inflammation and aging-related regulators were enriched in elderly skin. In our recently work (Zhang et al., 2021), the dermal ECM components of keloids tissues have been identified. By comparing the data with the keloids ECMs, we found some of the components, e.g., SLPI, THBS3, show both high expression trends in keloids and in elderly skin, indicating these ECMs could share similar functions in elderly and keloids skin tissue. For example, SLPI is a secretory inhibitor, which can promote the immune response by protecting the epithelial surface from the attack of endogenous proteolytic enzymes. Our results showed this protein was highly expressed in the elderly skin, which may lead to increasing immune response and aging of elderly skin. Thus, SLPI could be used as a potential marker of skin diseases with abnormal immune response.

## Conclusion

Our study is the first to use a decellularized method combined with proteomics tools to investigate the molecular differences and development characteristics of the dermal matrix during skin aging. The time-resolved ECM atlas constructed in this study identifies the ECM components with different types in tollder, teenager, adult and elderly, providing a new standard of age-specific skin ECM composition to discover new clues regarding tissue engineering for skin regeneration. In addition, the age-specific functions of different types of ECM proteins during skin aging were systematically analyzed, providing a comprehensive understanding of age-related changes in human skin ECM proteins and potential value in predicting other disease state in skin tissues.

## Data Availability

The datasets presented in this study can be found in online repositories. The names of the repository/repositories and accession number(s) can be found below: http://www.proteomexchange.org/, PXD016440.
