# Supplementary figures and images for: Time-Resolved Extracellular Matrix Atlas of the Developing Human Skin Dermis
Source: Front Cell Dev Biol. 2021 Nov 26;9:783456. doi: 10.3389/fcell.2021.783456 (PMC8661536; doi:10.3389/fcell.2021.783456)

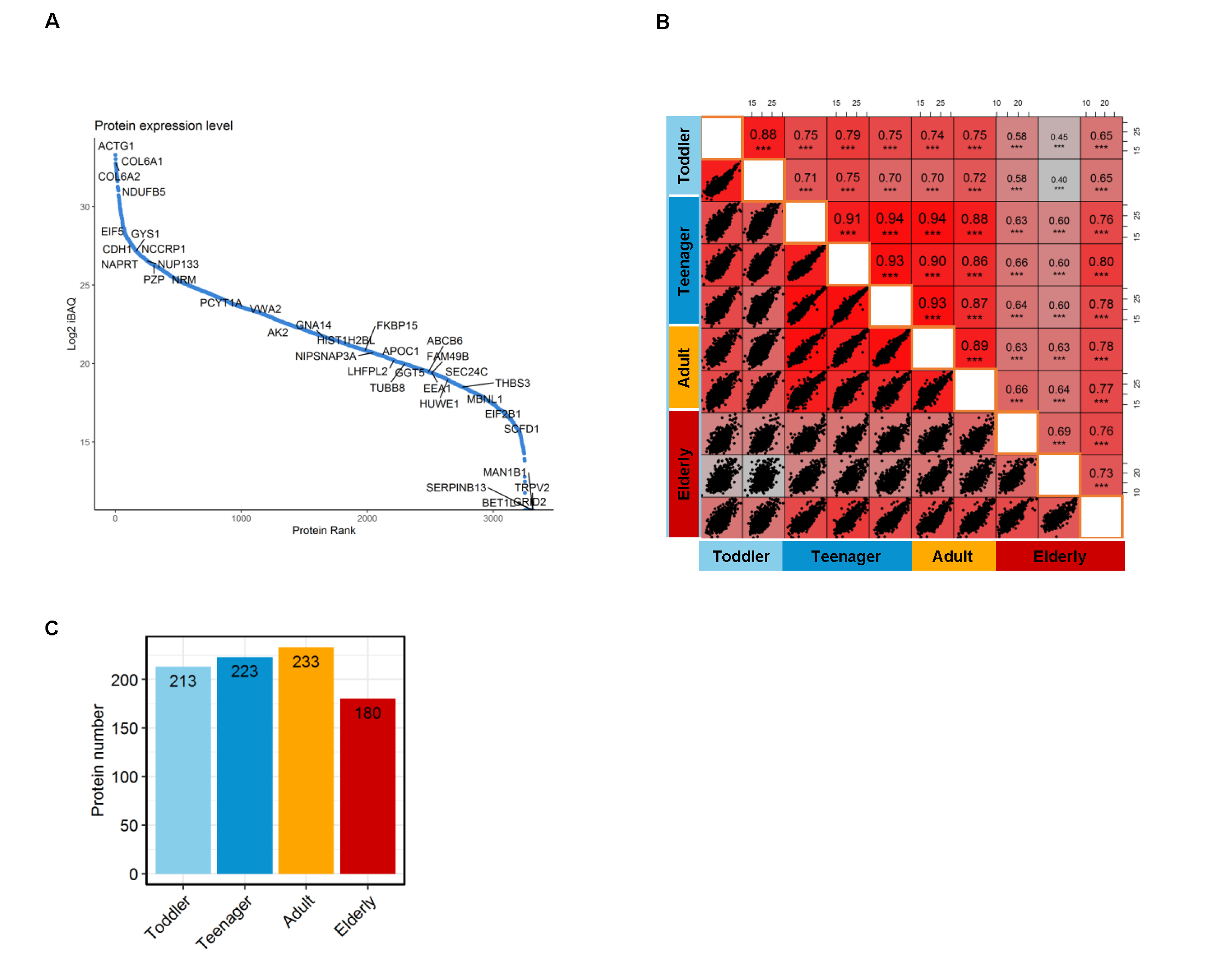

Supplement: Supplementary file 3 [file Presentation1.ZIP › Supplementary Figure 2.tiff]

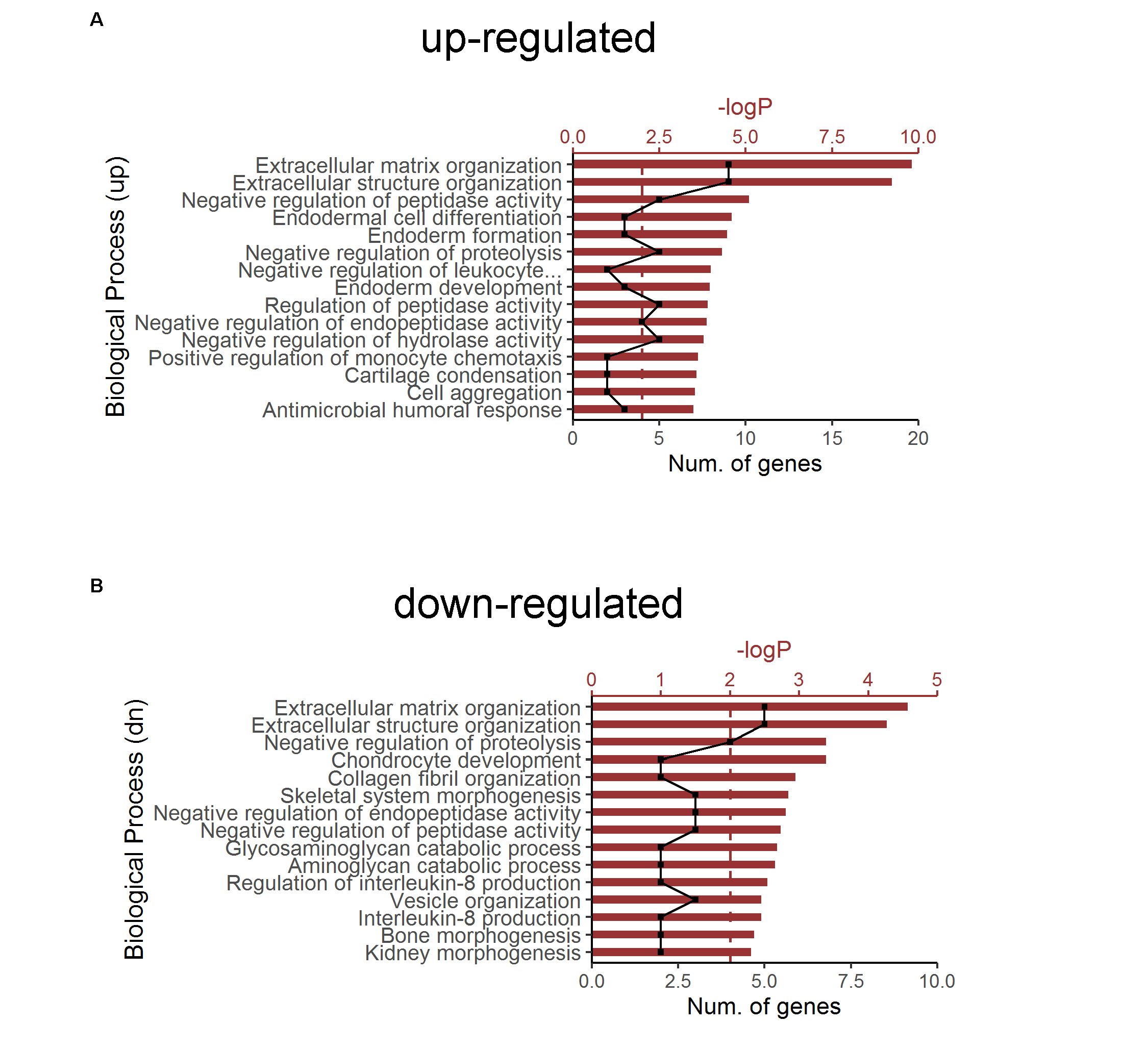

Supplement: Supplementary file 3 [file Presentation1.ZIP › Supplementary Figure 3.tiff]

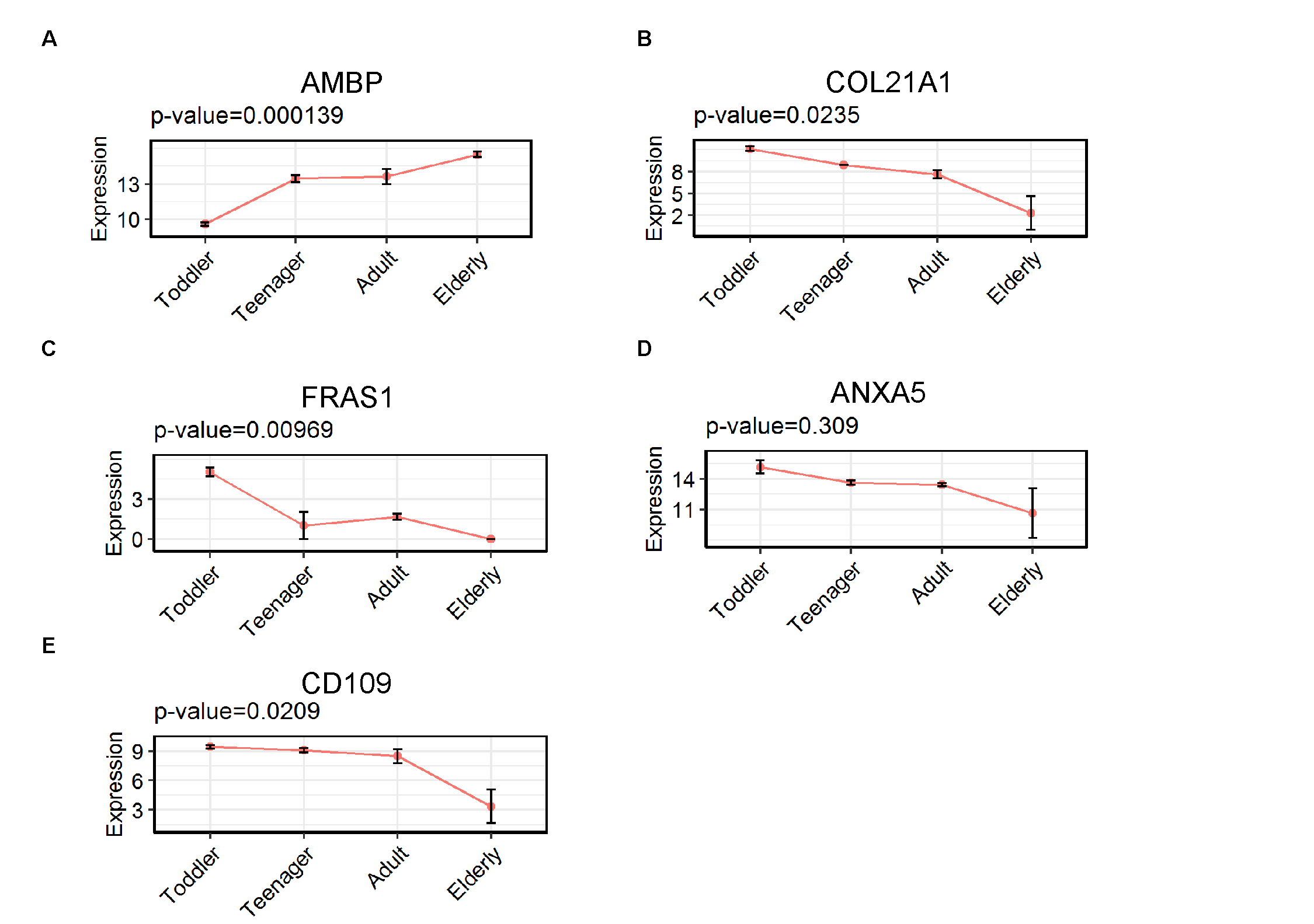

Supplement: Supplementary file 3 [file Presentation1.ZIP › Supplementary Figure 4.tiff]

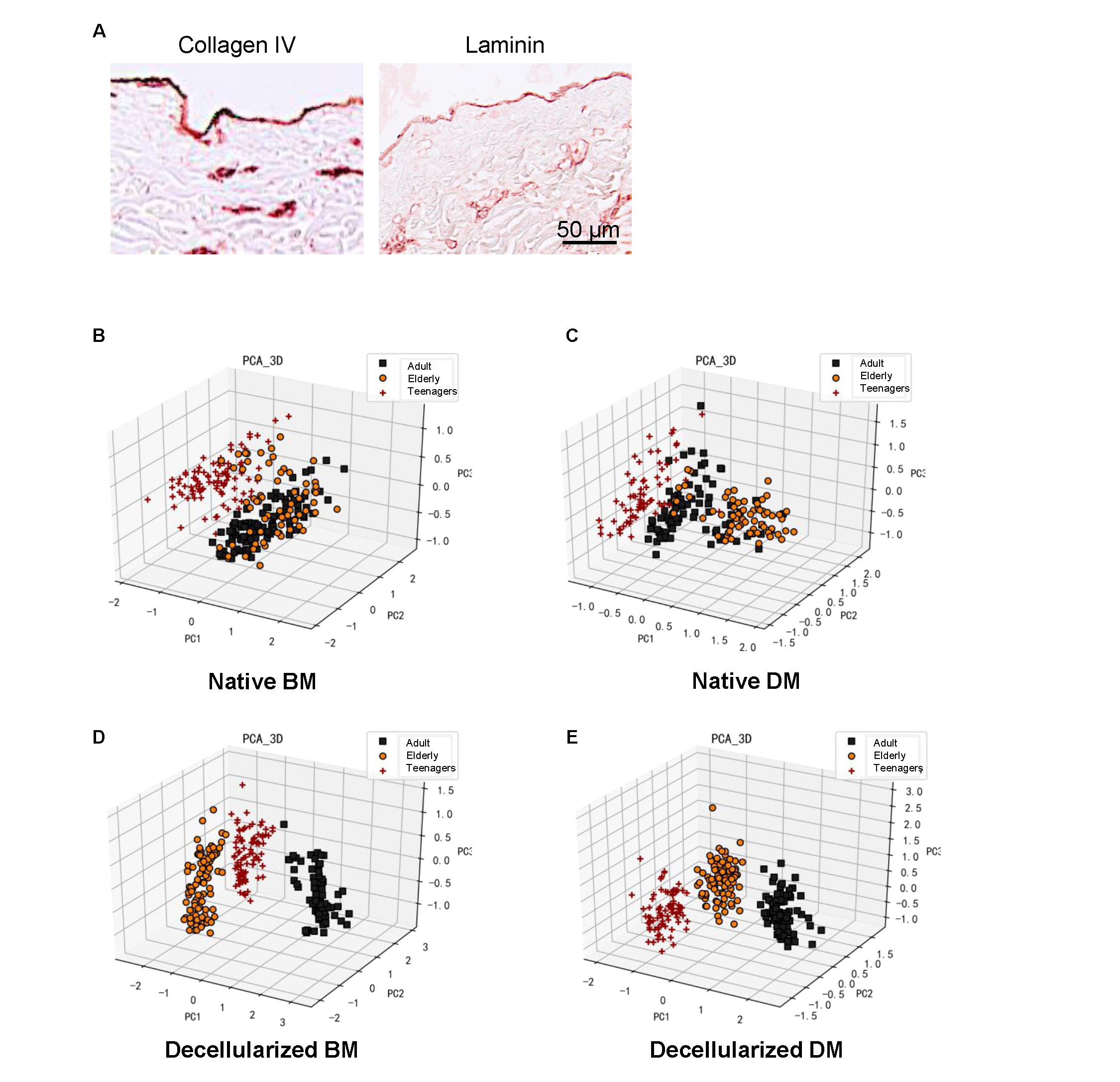

Supplement: Supplementary file 3 [file Presentation1.ZIP › Supplementary Figure 1.tiff]
